# Association Between Subclinical Hypothyroidism and the Prognosis of Diabetes Mellitus and Subsequent Complications: A Retrospective Cohort Study

**DOI:** 10.7759/cureus.48329

**Published:** 2023-11-05

**Authors:** Mariam Alharbi, Haifa N Alsaleem, Raghad Almuhaisni, Haytham S Alzeadi, Rana I Alsamani, Saleh I Alhammad, Abdulelah M Alharbi

**Affiliations:** 1 Internal Medicine, College of Medicine, Qassim University, Buraydah, SAU; 2 Medicine and Surgery, Qassim University, Buraydah, SAU

**Keywords:** metabolic disorders, tsh, hba1c, metformin, subclinical hypothyroidism, diabetes mellitus

## Abstract

Background

Diabetes mellitus (DM) and subclinical hypothyroidism (SCH) are prevalent metabolic and endocrine disorders. Previous studies have suggested potential associations between SCH and metabolic disorders, including DM. This study aimed to explore the relationship between SCH and DM in patients from the Qassim Region in Saudi Arabia.

Methods

A retrospective cohort study was conducted on patients who attended the Qassim University clinics from December 2017 to December 2021. Participants were divided into two groups: SCH patients without a history of DM and age- and sex-matched controls without SCH or DM. Parameters, such as DM occurrence, DM duration, glycosylated hemoglobin (HbA1c) values, and metformin use, were evaluated.

Results

The prevalence of DM was 70.4% in the SCH group and 83.3% in the non-SCH group, with no significant difference between the groups (p=0.203). The mean HbA1c values and DM duration showed no significant variations between the two groups. However, metformin use was significantly higher in the SCH group at 74.1% compared to 50.0% in the non-SCH group (p=0.047). No correlation was observed between HbA1c and thyroid-stimulating hormone (TSH) levels.

Conclusions

While the study indicates a potential association between SCH and DM management, especially with regard to metformin usage, there does not appear to be a significant relationship between SCH and DM development or progression in this population.

## Introduction

Diabetes mellitus (DM) is the most common metabolic disorder characterized by hyperglycemia resulting from inappropriate insulin secretion or resistance [1]. The prevalence of DM is increasing due to the increase in obesity, physical inactivity, population growth, aging, and urbanization [2]. Chronic uncontrolled DM has many long-term complications, including organ damage, vision loss, renal failure, and cardiovascular diseases [3].

Hypothyroidism occurs when the thyroid gland fails to produce sufficient thyroid-producing hormones, such as triiodothyronine (T3) and thyroxine (T4) [4]. Symptoms may include weight gain, cold intolerance, constipation, and proximal muscle weakness [5]. Subclinical hypothyroidism (SCH) is a mild form of hypothyroidism characterized by an elevated thyroid stimulating hormone (TSH) level with normal free T4 levels [6,7]. Thyroid disorders are prevalent in the general population and are the second most common condition affecting the endocrine system after DM [8].

Several studies suggested a relationship between SCH and metabolic disorders, especially DM, hypertension, and hypercholesterolemia [1,9-11]. Thyroid hormones and insulin are both critical in cellular metabolism [12]; any change in one of them can result in functional derangement of the other [8,13]. This relationship between DM on and the thyroid can be seen at many levels, either at the hypothalamic and control of TSH release or at the conversion of T4 to T3 [8]. It was suggested that the association between SCH and DM is resulting from the stimulating carbohydrate metabolism by tyrosine-based hormones via multiple mechanisms, including insulin-induced glucose uptake into cells and the enhancement of gluconeogenesis [14].

Despite the large body of evidence supporting the association between thyroid dysfunction and DM, the association between SCH and DM is debatable. Some studies showed a significant association [1,15], while others could not find any association [16-18], highlighting the need for further studies. Therefore, this study aimed to investigate the association between SCH and DM among a group of who presented to the Qassim University clinics, Qassim Region in Saudi Arabia.

## Materials and methods

Study design and settings

We conducted a retrospective cohort study on patients who presented to the Qassim University clinics, Qassim Region in Saudi Arabia, from December 2017 to December 2021. The study enrolled two groups: patients with laboratory and clinically confirmed SCH at the index date of the study with no history of DM and patients without SCH or DM at the index date. Both groups were age- and sex-matched. The patients’ records were historically followed to identify the occurrence of DM. Written informed consent was obtained from all participants. The study protocol was approved by the ethics committee of the Qassim University and the Saudi Ministry of Health (IRB: 21-07-12).

Population

We included adults (≥18 years old) patients with confirmed diagnosis of SCH at the index date, December 2017, with no history of DM. The comparison cohort was selected randomly from patients who visited the Qassim University clinics with no history of SCH of DM and were age- and sex-matched to the SCH patients. We excluded patients who had chronic renal failure or glomerulonephritis, liver dysfunction, severe infection, malignant tumor, pregnancy, uncontrolled hypertension, congestive heart failure, received contraceptives or hormone replacement therapy, and amiodarone or lithium preparations. 

Data collection 

Patient baseline data were obtained from the electronic medical record system based on age, gender, body mass index (BMI), blood pressure, history of hypertension, duration of SCH, glycosylated hemoglobin (HbA1c), thyroxine (FT4), tri-iodothyronine (FT3), and TSH. The diagnosis of DM was based on the WHO criteria [19]. In terms of SCH, patients were defined as persistently elevated TSH values (at least twice and at least three months apart) with FT4 levels within the reference range [7,20]. Data were securely stored and maintained with strict confidentiality. 

Statistical analysis 

The sample size was determined using a specific statistical calculating formula. The data were presented using numbers and percentages for all categorical variables, whereas continuous variables were summarized as mean and standard deviation. A chi-square test was used to measure the association between SCH concerning the baseline and clinical characteristics of the patients. A p-value of less than 0.05 was taken as the significant level for all statistical tests. All statistical analyses were done using IBM SPSS Statistics for Windows, version 26 (released 2019; IBM Corp., Armonk, New York, United States.

## Results

Demographic characteristics

The mean age for those with SCH was 54.3±14.1 years and 54.6±15.0 years for those without SCH (p=0.975). For education, 22.2% of the SCH group had no formal education compared to 23.8% in the non-SCH group (p=0.438). For nationality, 92.6% of the SCH group were Saudis, which was almost identical to the 92.9% in the non-SCH group (p=0.967). A notable difference was observed in the family history of SCH: 51.9% of the SCH group had a family history of SCH, significantly higher than the 26.2% in the non-SCH group (p=0.030). On the other hand, 11.1% of the SCH group had a family history of DM compared to 26.2% in the non-SCH group (p=0.128). The mean duration of SCH was 5.19±5.47 years and the mean dose of thyroxine was 0.075±0.039 mg, as shown in Table 1.

**Table 1:** Demographic and clinical characteristics SCH: subclinical hypothyroidism, non-SCH: non-subclinical hypothyroidism, DM: diabetes mellitus, TSH: thyroid stimulating hormone

Variables		SCH (n= 27)	Non-SCH (n= 42)	p-value
Age, years		54.3±14.1	54.6±15.0	0.975
Education	None	6 (22.2%)	10 (23.8%)	0.438
	Diploma or lower	12 (44.4%)	11 (26.2%)	
	Collage students	6 (22.2%)	14 (33.3%)	
	Postgraduate	3 (11.1%)	7 (16.7%)	
Nationality	Saudi	25 (92.6%)	39 (92.9%)	0.967
	Others	2 (7.4%)	3 (7.1%)	
Family history of SCH	No	13 (48.1%)	31 (73.8%)	0.030
	Yes	14 (51.9%)	11 (26.2%)	
Family history of DM	No	24 (88.9%)	31 (73.8%)	0.128
	Yes	3 (11.1%)	11 (26.2%)	
Disease duration, years		5.19±5.47	-	-
Thyroxine dose, mg		0.075±0.039	-	-
TSH level		10.3±7.24	-	-

Association between SCH and DM

In a comparison between the SCH and non-SCH groups in Table 2, the distribution of participants with DM showed that 70.4% of those in the SCH group and 83.3% in the non-SCH group had DM, with no statistically significant difference (p=0.203). When evaluating the DM duration, individuals with less than five years of DM constituted 78.9% in the SCH group compared to 48.6% in the non-SCH group (p=0.080). The mean HbA1c values were 6.76±1.15% for the SCH group and 6.89±0.96% for the non-SCH group (p=0.272). A total of 48.1% in the SCH group and 35.0% in the non-SCH group had HbA1c levels below 6.5% (p=0.282). As for metformin use, 74.1% of the SCH group and 50.0% of the non-SCH group were on metformin, with this difference being statistically significant (p=0.047). Lastly, 18.5% of participants in the SCH group had DM-related complications in contrast to 33.3% in the non-SCH group, but this difference was not statistically significant (p=0.179). No significant correlation was found between HbA1c and the TSH level (r=0.013, p=0.947).

**Table 2:** Comparison between SCH and non-SCH groups SCH: subclinical hypothyroidism, non-SCH: non-subclinical hypothyroidism, DM: diabetes mellitus, HBA1C: glycated hemoglobin

Variables		SCH (n= 27)	Non-SCH (n= 42)	p-value
DM	No	8 (29.6%)	7 (16.7%)	0.203
	Yes	19 (70.4%)	35 (83.3%)	
DM duration	<5 years	15 (78.9%)	17 (48.6%)	0.080
	5-10 years	3 (15.8%)	10 (28.6%)	
	>10 years	1 (5.3%)	8 (22.9%)	
HbA1c (%)		6.76±1.15	6.89±0.96	0.272
HbA1c level	<6.5%	13 (48.1%)	14 (35.0%)	0.282
	≥6.5%	14 (51.9%)	26 (65.0%)	
Metformin use	No	7 (25.9%)	21 (50.0%)	0.047
	Yes	20 (74.1%)	21 (50.0%)	
DM complications	No	22 (81.5%)	28 (66.7%)	0.179
	Yes	5 (18.5%)	14 (33.3%)	

## Discussion

The study found that the prevalence of DM was slightly higher in the non-SCH group than in the SCH group. While the duration of DM and HbA1c values showed some variation between the two groups, the only statistically significant difference observed was in the use of metformin, which was more prevalent in the SCH group. Clinically, these findings suggest that there might be an association between SCH and DM management, particularly concerning metformin use. However, there was no significant association between DM development and progression. 

In our investigation, we adopted an alternate approach by evaluating the prevalence of DM among patients with SCH. This contrasts with the majority of preceding studies that primarily sought to determine the prevalence of SCH among DM patients. This shift in perspective provides a unique lens through which we can better understand the interrelationship between DM and SCH. Whereas traditionally, the emphasis has been on understanding how often diabetic patients may present with SCH, our approach offers insights into how frequently those with SCH may be at risk for or exhibit DM. This methodological distinction underscores the importance of comprehensive studies in both directions to develop a more holistic understanding of the coexistence and potential interplay between these two conditions.

In this study, the overall prevalence of DM among patients with SCH was 70.4%. The majority of related studies aimed to identify the prevalence of SCH among diabetic patients. A previous meta-analysis of 36 articles showed that the prevalence of SCH among patients with DM was 12% (95% confidence interval (CI): 10% to 14%) [1]. In addition, studies reported that the prevalence of SCH among diabetic patients ranges from 1.6% to 29.8% [21-27]. The high prevalence of DM in our patients is alarming, which may hinder their management. While a subset of diabetic patients may develop SCH, a considerably larger proportion of those with SCH might be at risk for or already have DM, which highlights the need for reciprocal investigations to understand the intricate relationship and potential bidirectional risks between DM and SCH.

In our study, the relationship between SCH and various DM indicators, namely, prevalence, HbA1c levels, and DM complications, did not yield a significant association. This outcome contrasts with the bulk of existing research, where a significant association between SCH and these diabetes-related parameters is often emphasized. A meta-analysis showed that the risk of developing SCH among diabetic patients was significantly higher than non-diabetic (odds ratio (OR) = 2.32, 95% CI: 1.97 to 2.73) [1]. Another meta-analysis showed that SCH with positive antithyroid autoantibodies significantly increased the risk of DM (OR = 3.22, 95% CI: 1.72 to 6.03) [28]. However, there are some studies that could not find a significant association between SCH and DM. Zhu et al. and Zhang et al. showed that the risk of SCH was comparable among diabetic and non-diabetic patients (OR = 2.90, 95% CI: 0.85 to 9.89 and OR = 4.14, 95% CI: 0.92 to 18.76, respectively) [1,15-30]. It is plausible that differences in study populations, methodologies, or even regional factors could contribute to these divergent findings. However, it is also worth noting that while research can provide general trends and associations, individual studies may still yield unique outcomes due to a myriad of factors. 

Interestingly, our study showed a significant trend in medication use: a higher proportion of patients with SCH was prescribed metformin compared to their non-SCH counterparts. This observation could suggest that there might be underlying factors or conditions making those with SCH more amenable or requiring of Metformin treatment. It may also point toward different treatment protocols or perceptions among healthcare professionals regarding the management of diabetic patients with SCH. While the reason for this trend remains a subject for further investigation, the finding emphasizes the importance of understanding not just disease associations but also treatment patterns and their potential implications.

Our results revealed that TSH did not show a significant correlation with HbA1c (p = 0.947). This is almost consistent with the report of Chutia et al., who found a positive but insignificant association between TSH and insulin resistance among diabetic patients [25]. Meanwhile, Shabnam et al. documented a significant association between fasting plasma glucose and serum TSH levels [26], while Hendarto et al. found a significant correlation between HbA1c and SCH in T2DM patients [30].

It is important to note that approximately 27.5% of our patients developed complications due to their comorbid conditions, with cataracts being the most recognized complication. Other complications were decreased vision and retinopathy. However, our results indicate that complication was not a relevant factor of SCH. This is consistent with the study of Khassawneh et al., wherein thyroid disorders had no significant association with complications or duration of diabetes [31]. On the contrary, Han et al. documented an association between SCH and diabetic complications [1]. In detail, their observation shows that SCH might increase the development of diabetic complications, with an overall OR of 1.87 for diabetic peripheral neuropathy, 1.85 for peripheral arterial disease, 1.74 for diabetic nephropathy, and 1.42 for diabetic retinopathy [1]. This has been concurred by the study of Furukawa et al. [32]. According to their multivariate regression model, they noted that diabetic nephropathy greatly influences SCH, hypertension, and smoking [32].

Clinical implications and future directions 

The findings from our study add a distinctive layer to the evolving landscape of understanding between SCH and DM. Our emphasis on gauging the prevalence of DM among those with SCH, rather than the more common reverse approach, unravels a substantial rate of coexistence. Despite this, the clinical indicators of DM - from its onset to progression marked by HbA1c levels and complications - did not show a significant association with SCH. This disparity with existing literature, where diabetes often correlates with SCH, could be attributed to the small sample of this study or the heterogeneity of clinical experiences across populations. Such discrepancies could arise from demographic nuances, variances in methodologies, or regional healthcare patterns. These differing results accentuate the necessity for more expansive studies that can bridge such gaps. As we move forward, a deeper dive into understanding the intrinsic mechanisms connecting SCH and DM, buttressed by both our findings and those from other global studies, can pave the way for more effective patient-centric clinical approaches and interventions.

Limitations

Our study has several inherent limitations that warrant consideration. First, being a retrospective cohort design, it is susceptible to biases associated with retrospective data collection, including potential missing data or inconsistencies in documentation. The sample size of our study is relatively small, which may limit the statistical power to detect subtle differences and associations. A larger sample could have potentially provided more robust and generalizable results. Furthermore, our study was conducted at a single center, which may not be representative of the broader population or other clinical settings. This single-center nature raises concerns about the external validity of our findings, making it challenging to extrapolate our results to other regions or diverse patient groups. In addition, potential confounding factors that were not accounted for could influence the results. Future studies with a multi-center approach, larger sample size, and more comprehensive data collection methods would be beneficial to corroborate and expand upon our findings.

## Conclusions

This study shows that while the prevalence of DM was slightly higher in the non-SCH group, the notable finding was the significantly higher use of metformin among SCH patients compared to their non-SCH counterparts. Although there were variations in DM duration and HbA1c values between the two groups, these were not statistically significant. The research also revealed that TSH levels did not significantly correlate with HbA1c levels. The study suggests that while there may be an association between SCH and DM management, particularly concerning metformin use, there is no significant link between SCH and DM development or progression.
